# Delayed Conversion of a Fibroadenoma Into a Large Phyllodes Tumor: A Case Report

**DOI:** 10.7759/cureus.30795

**Published:** 2022-10-28

**Authors:** Thomas C Hartmann, Mariam W Hanna

**Affiliations:** 1 Radiology, University of Central Florida College of Medicine, Orlando, USA; 2 Radiology: Breast Imaging, University of Florida, Gainesville, USA

**Keywords:** med12, breast mass, breast neoplasm, fibroadenoma, phyllodes tumor

## Abstract

Phyllodes tumors are rare breast neoplasms that share many clinical characteristics with fibroadenomas, their benign counterpart. Despite their shared features, few reports have been made about the potential mechanisms by which a fibroadenoma can convert into a phyllodes tumor. This case report describes a large phyllodes tumor presenting in a 50-year-old female patient with a history of an excised biopsy-proven fibroadenoma eight years prior. Here, we exhibit our imaging findings and discuss plausible mechanisms for the conversion of a fibroadenoma into a phyllodes tumor, including genomic alterations.

## Introduction

Fibroadenomas and phyllodes tumors are part of a category of biphasic breast masses called cellular fibroepithelial lesions (CFLs). They are termed biphasic because they consist of both stromal and epithelial components [1]. Due to this similarity in composition, these masses can be difficult to distinguish between using routine breast imaging techniques such as mammography and ultrasound.

Core needle biopsy under imaging guidance is the current standard of care for the diagnosis of CFLs [2]. Yet, the test sensitivity in distinguishing between a fibroadenoma and phyllodes tumor on tissue pathology is hindered by their similar heterogeneous structures and variability in stromal cellularity. Histologically, a phyllodes tumor can be accompanied by a unique intracanalicular growth pattern with “leaf-like” stromal proliferations, but this finding is not always well-defined [3]. In addition, depending upon the biopsy location, a definitive phyllodes tumor may have more features consistent with a fibroadenoma, and vice versa, leading to a potential misdiagnosis.

The distinction between a fibroadenoma and a phyllodes tumor is clinically significant for determining management. Fibroadenomas may be safely followed with routine screening, as they tend to fluctuate in size based on hormonal patterns. In contrast, phyllodes tumors rarely regress and therefore require operative management due to their risk for rapid, uncontrolled growth. For definitive treatment, phyllodes tumors undergo surgical excision with wide margins (> 1 cm), requiring a significant amount of healthy breast tissue to be sacrificed [4].

In recent years, literature has suggested that a common genetic predisposition may be responsible for the development of a phyllodes tumor from a fibroadenoma, such as mutations in Mediator Complex Subunit 12 (*MED12*) exon 2, Telomerase Reverse Transcriptase (*TERT*) promoter, and Retinoic Acid Receptor Alpha (*RARA*), which are important genes for DNA synthesis. However, the mechanism remains unclear [5,6]. This would be consistent with the similar biological and histological features seen between these masses, especially early on in their disease course. In this case report, we discuss a patient who developed a large phyllodes tumor after having a biopsy-proven fibroadenoma excised at the same location eight years prior.

## Case presentation

**Figure 1:**
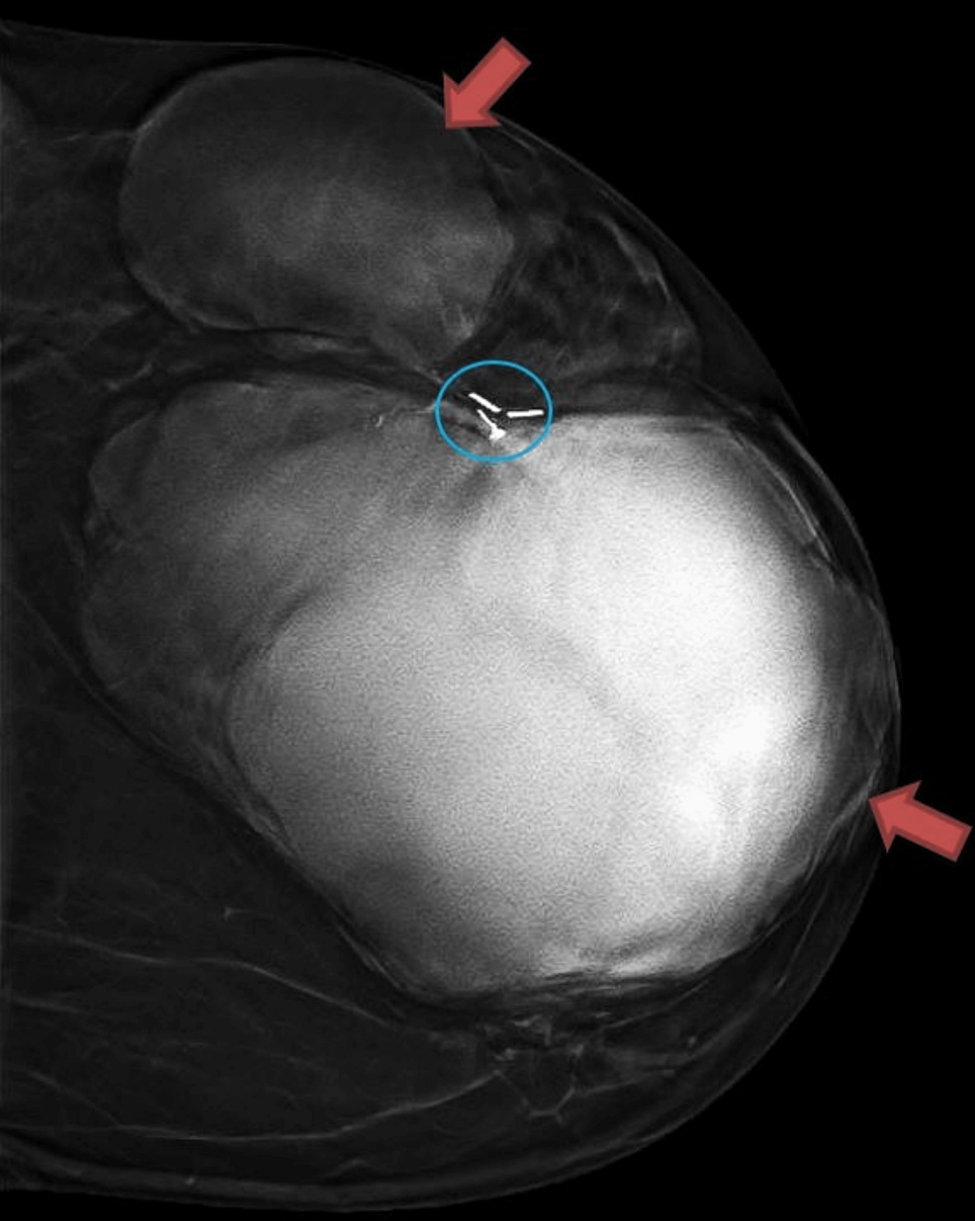
Left CC mammogram showing two large multi-lobulated masses measuring 15.8 x 12.3cm and 5.0 x 8.3cm (red arrows) The biopsy marker clip seen between the two masses is consistent with the patient’s history of prior excisional biopsy in 2014 (blue circle).

**Figure 2:**
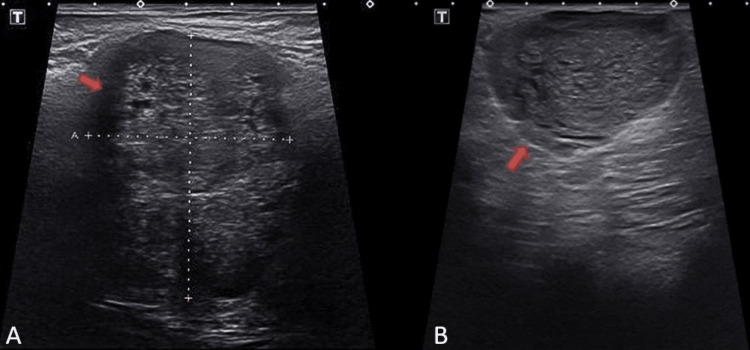
Ultrasound of the left breast indicative of two large masses (red arrows) at the A) 12:00 position and B) 3:00 position, 8 cm from the nipple

In June 2022, a 50-year-old female presented to the outpatient diagnostic imaging clinic after presenting to the emergency department a month prior for breast pain secondary to multiple, palpable left breast masses. The patient continued to endorse left breast pain at this time as well as mild left breast engorgement. A review of symptoms was significant for chills, fatigue, and unexplained weight loss. On breast exam, there was noticeable asymmetry, with the left breast greater in size than the right. The left breast exam was significant for nipple eversion and multiple large tender palpable masses encompassing a majority of the breast tissue. The right breast displayed no abnormalities. The patient’s past medical history was significant for an excisional biopsy of a pathology-proven benign fibroadenoma eight years ago. The patient was recommended for mammography and ultrasound (Figures 1-2).

**Figure 3:**
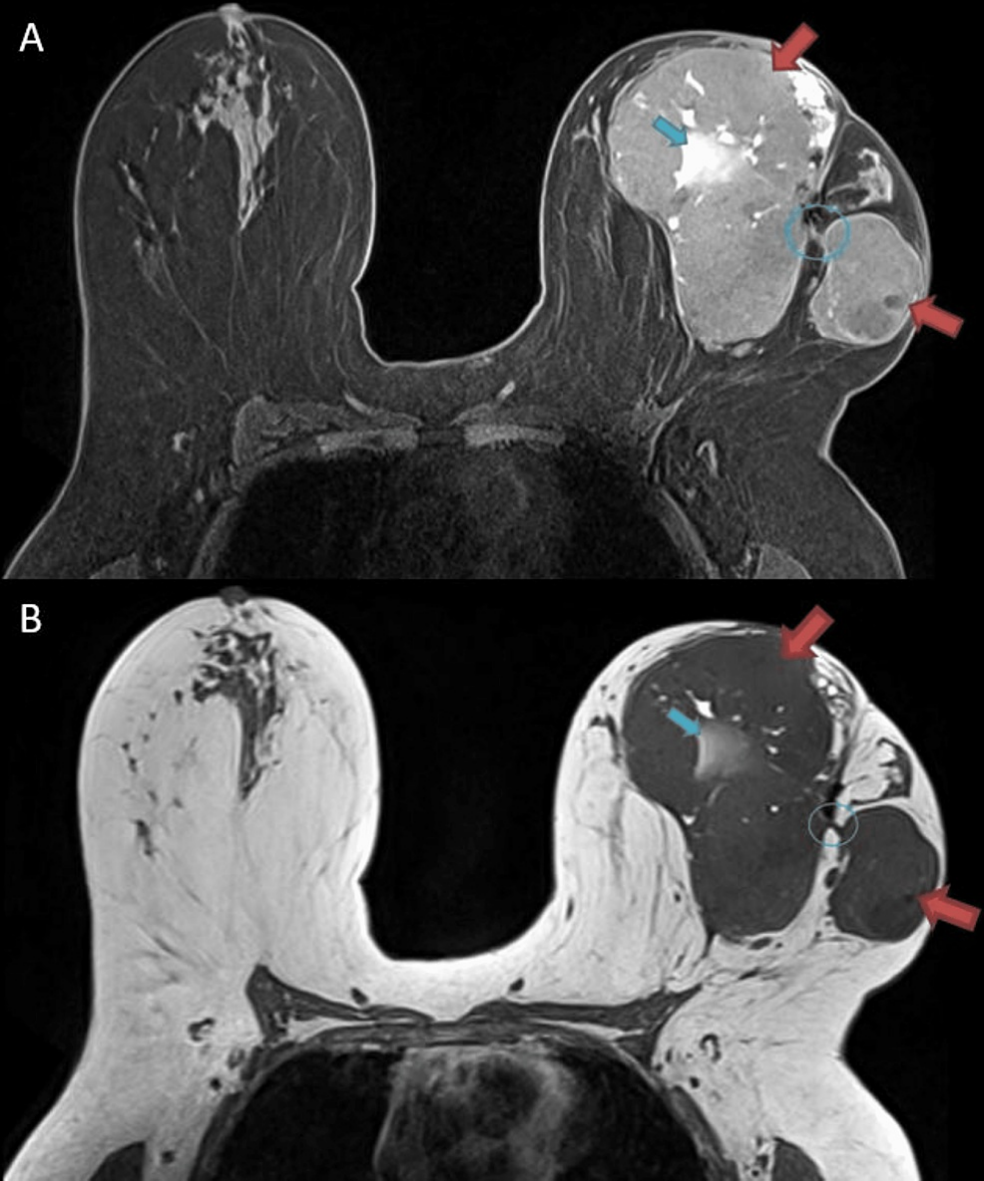
A) T2-weighted and B) T1-weighted MRI of the left breast was significant for two heterogeneously enhancing masses interconnected at the site of the prior surgical scar and clip representing a single process (red arrows; blue circle) The conglomeration measures 13.0 x 15.0 x 14.0 cm (AP × TR × CC). There is apparent, necrosis within the dominant portion of the lesion (blue arrow).

Mammography and ultrasound findings were suggestive of a potential malignancy (breast imaging-reporting and data system (BI-RADS) = 4). The patient was recommended for an ultrasound-guided, vacuum-assisted core needle biopsy that demonstrated a cellular fibroepithelial lesion with atypical ductal hyperplasia suggestive of a large phyllodes tumor. The patient was referred to the breast surgery service, which suggested performing a pre-operative breast MRI (Figure 3).

**Figure 4:**
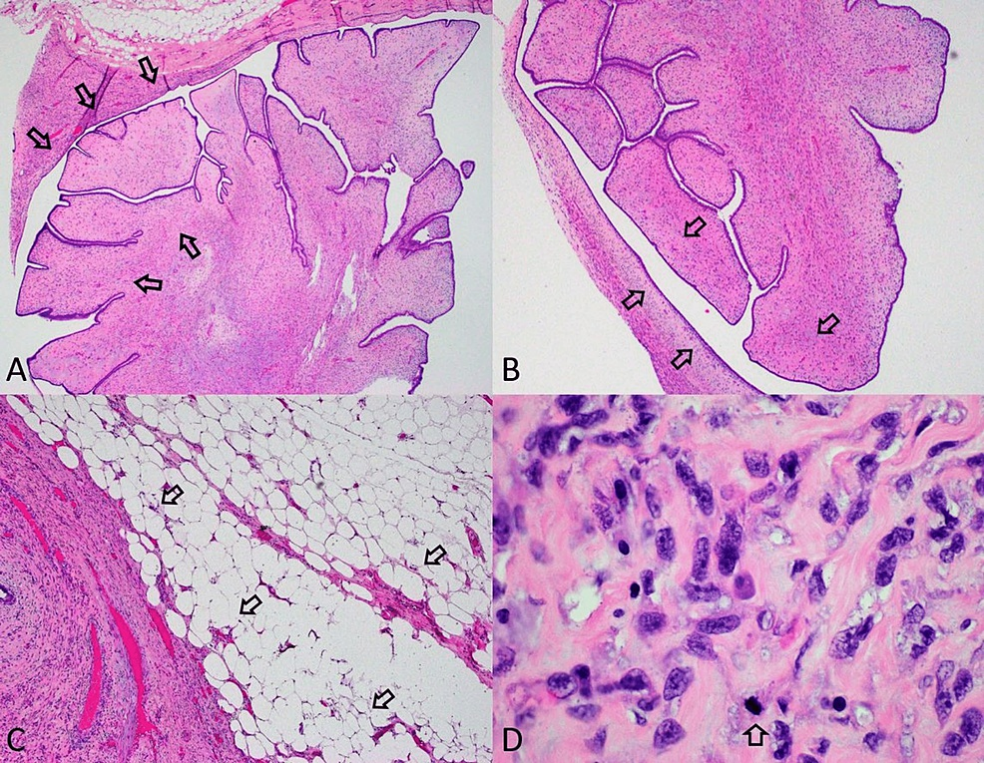
A-B) Histopathology shows stromal proliferation of leaf-like patterns with mild stromal expansion and moderate cellularity (black arrows). C) Lesion demonstrates focal infiltrative borders involving the surrounding adipose tissue (black arrows). D) Cytology shows nuclear pleomorphism with marked stromal atypia and scattered mitotic figures (4 to 5/10 HPF) (black arrow)

A simple left-sided mastectomy was then performed with wide-margin resection of the gross specimen. Histopathology showed a focally infiltrative lesion with stromal proliferation featuring areas of leaf-like pattern and moderate cellularity (Figures 4A-4C). The cytology was performed which showed nuclear pleomorphism with mild to moderate stromal atypia and scattered mitotic figures (4 to 5/10 HPF) (Figure 4D). The final diagnosis was a large, borderline phyllodes tumor measuring 15 cm in its maximal dimension and weighing 1.9 kg (4.2 lbs).

## Discussion

We report a unique case of a large, borderline phyllodes tumor presenting in a 50-year-old female who had a prior surgical excision of a biopsy-proven fibroadenoma in the same location. Few cases have been reported in the literature and after an extensive literature review, we believe the patient in this case to be one of the first reported where a prior excised fibroadenoma is now re-occurring as a large phyllodes tumor after a significant time lapse.

The etiology of this patient’s large phyllodes tumor has several possibilities. First, we cannot rule out misdiagnosis of the patient’s previously excised fibroadenoma actually being a phyllodes tumor due to their overlapping histological features. However, literature has shown that when phyllodes tumors do recur, it typically happens within two years of the initial excision [7]. In this patient’s case where there were eight years between mass reoccurrence, this theory may be less likely.

The more interesting etiology is the transformation of a fibroadenoma into a now large phyllodes tumor after an eight-year time lapse. For comparison, we identified three other transformation cases currently published in the literature where a biopsy-proven fibroadenoma had later converted into a phyllodes tumor. The first case we identified described an 18-year-old female who had a relapsing juvenile fibroadenoma excised via lumpectomy. Despite surgical removal with sufficient margins, the breast lesion progressively recurred into a borderline phyllodes tumor over a one-year time period [8]. Another case was seen in a 40-year-old female who developed a malignant phyllodes tumor from a fibroadenoma over the course of three years, despite minimal changes in the overall breast mass size [9]. The last case we identified highlighted a 27-year-old female who had two prior fibroadenomas removed via partial mastectomies. Five years later, the patient presented with two gradually enlarging breast masses in the same breast, later diagnosed as malignant phyllodes tumors [10]. Despite the similarities between these cases and the one presented in our case report, one interesting point should be noted about the timeline over which these masses developed. To the best of our knowledge, we believe our case to be the longest time-lapse (8 years) for the conversion of a fibroadenoma into a phyllodes tumor.

A parallel between our case report and the ones previously discussed is the curiosity to discover a link between these breast fibroepithelial lesions. Recent studies have investigated mechanisms by which a fibroadenoma could develop into a phyllodes tumor, and therefore allow clinicians to better anticipate their potential for transformation. One area of promising research has been with genetics, as phyllodes tumors have already shown an association with other conditions such as Li-Fraumeni syndrome and BRCA1/2 mutations [11]. More specific to our case, recent literature has identified overlapping mutations associated with both fibroadenomas and phyllodes tumors, primarily MED12 exon 2, *TERT *promoter, and *RARA *genes [12]. Biologically, these are highly regulated genes involved in the DNA synthesis process, which, when mutated, can lead to the uncontrollable cell growth characteristic of these breast lesions. One study, in particular, looked at a variety of genetic mutations (including MED12 exon 2, *TERT *promoter, and *RARA*) and developed a novel 16-gene panel as a diagnostic tool for characterizing breast fibroepithelial lesions. After testing 275 breast specimens, Sim et al. recognized the MED12 exon 2 gene as the only mutation that frequently occurred in both fibroadenomas and phyllodes tumors at all grades, which they suggested meant a biological link existed between the two masses. In addition, the researchers found a statistically significant higher mutation rate of the *TERT* promoter and *RARA* gene in phyllodes tumors compared to fibroadenomas. Therefore, they concluded their 16-gene panel could potentially be used as an adjunctive diagnostic tool for distinguishing between fibroadenomas and phyllodes tumors on core biopsy [5].

## Conclusions

This case report highlights the potential for conversion of a fibroadenoma into a large phyllodes tumor, even after an eight-year time period. Both symptomatic fibroadenomas and phyllodes tumors are managed surgically, yet the lack of adequate margins on excision due to a confounding diagnosis can lead to additional future surgeries and loss of additional healthy breast tissue. Further research is required to determine if additional testing should be employed to better anticipate these breast lesion transformations. One area of promising research is with genomic testing, such as MED12 exon 2, promoter *TERT*, and *RARA *gene mutations, but despite promising research, literature shows it to add an indeterminate value in the clinical setting at this time.
